# Systemic Effects of Ulna Loading in Male Rats During Functional Adaptation

**DOI:** 10.1002/jbmr.101

**Published:** 2010-04-01

**Authors:** Susannah J Sample, Ryan J Collins, Aliya P Wilson, Molly A Racette, Mary Behan, Mark D Markel, Vicki L Kalscheur, Zhengling Hao, Peter Muir

**Affiliations:** 1 Comparative Orthopaedic Research Laboratory, School of Veterinary Medicine, University of Wisconsin–Madison Madison, WI, USA; 2 Department of Comparative Biosciences, School of Veterinary Medicine, University of Wisconsin–Madison Madison, WI, USA; 3 Department of Surgical Sciences, School of Veterinary Medicine, University of Wisconsin–Madison Madison, WI, USA; 4 Department of Medical Sciences, School of Veterinary Medicine, University of Wisconsin–Madison Madison, WI, USA

**Keywords:** functional adaptation, mechanical loading, brachial plexus anesthesia, thoracic limb, pelvic limb, TNF-α, RANKL, OPG, TRAP5b

## Abstract

Functional skeletal adaptation is thought to be a local phenomenon controlled by osteoctyes. However, the nervous system also may have regulatory effects on adaptation. The aim of this study was to determine the effects of loading of a single bone on adaptation of other appendicular long bones and whether these responses were neuronally regulated. Young male Sprague-Dawley rats were used. The right ulna was loaded to induce a modeling response. In other rats, a second regimen was used to induce bone fatigue with a mixed modeling/remodeling response; a proportion of rats from each group received brachial plexus anesthesia to induce temporary neuronal blocking during bone loading. Sham groups were included. Left and right long bones (ulna, humerus, tibia, and femur) from each rat were examined histologically 10 days after loading. In fatigue- and sham-loaded animals, blood plasma concentrations of TNF-α, RANKL, OPG, and TRAP5b were determined. We found that loading the right ulna induced an increase in bone formation in distant long bones that were not loaded and that this effect was neuronally regulated. Distant effects were most evident in the rats that received loading without bone fatigue. In the fatigue-loaded animals, neuronal blocking induced a significant decrease in plasma TRAP5b at 10 days. Histologically, bone resorption was increased in both loaded and contralateral ulnas in fatigue-loaded rats and was not significantly blocked by brachial plexus anesthesia. In young, growing male rats we conclude that ulna loading induced increased bone formation in multiple bones. Systemic adaptation effects were, at least in part, neuronally regulated. © 2010 American Society for Bone and Mineral Research.

## Introduction

The physiologic mechanisms that regulate functional adaptation of the skeleton are not well defined despite intense interest in this field. Although the skeleton is composed of a large number of bones, little is known about the potential for crosstalk between bones. The failure of functional adaptation to protect the skeleton from fracture is an important clinical problem, particularly as it affects the elderly and athletes.(1,2) Modeling and remodeling of bone are the means by which adaptation optimizes bone mass within the skeleton.(3,4) A large proportion of remodeling is thought to be targeted to the repair of bone microdamage.(4)

The ability of bone to sense changes in strain associated with biophysical loading events generally is considered a local phenomenon; only loaded bones undergo adaptation, with osteocytes and their dendritic processes acting to detect loading events.(5,6) Since work performed using a rabbit bone-loading model in 1971 suggested that adaptation in intact and “denervated” limbs was similar,(7) the possibility that additional physiologic pathways might regulate functional adaptation has received little scientific consideration. However, recent research has begun to challenge the paradigm that only loaded bones undergo adaptation. For example, mechanical loading of the proximal epiphysis of the tibia in a mouse model enhances load-dependent bone formation in the mid-diaphysis, where in situ strains are not altered by loading,(8) suggesting that crosstalk between different regions of the same bone can occur during adaptation. This concept was extended by work from our laboratory that suggests that skeletal responses to bone loading include changes in bone formation at distant skeletal sites that were not loaded.(9) By use of temporary brachial plexus anesthesia to block neuronal signaling between the loaded bone and the spinal cord, this work also suggests that bone formation in both loaded and contralateral nonloaded bones is, at least in part, neuronally regulated.(9) However, these studies were limited to thoracic limb long bones, and in other models, such as the mouse tibial loading model, similar effects have not been identified.(10)

The periosteum is the skeletal tissue with the greatest density of sensory nerve fibers,(11) which are arranged in a dense netlike meshwork that is optimized for detection of mechanical distortion.(12) Nerve branches or single neurons enter the bone cortex, often in association with the microvasculature, and connect individual bone cells to the central nervous system via unmyelinated sensory neurons.(13,14) Mechanical loading of bone induces plasticity in the sensory input to the central nervous system and enhances the connectivity of neural circuits between limbs, most likely via propriospinal pathways in the spinal cord.(15) Neural circuits potentially enable crosstalk among all four limbs.(15)

Taken together, these recent observations suggest the existence of a neuronally mediated physiologic system that may have important regulatory effects on functional adaptation of the skeleton. Additionally, the loading environment of a single long bone may influence adaptation of long bones throughout the skeleton. The purpose of this study was to determine whether the systemic effects of a single bout of cyclic bone loading, with and without induction of bone fatigue, involved appendicular long bones in both pelvic and thoracic limbs. Using the rat ulna end-loading model combined with brachial plexus anesthesia to temporarily block peripheral neuronal signaling in the loaded limb during the loading event,(9) we confirmed that unilateral ulna loading modulates bone formation in all limbs through a neuronal mechanism.

## Materials and Methods

### Animals

A homogeneous group of 52 young male Sprague-Dawley rats (body weight 290 to 305 g, age 65 to 81 days) was used for this study. Rats were provided with food and water *ad libitum*. All procedures were performed in accordance with guidelines of the American Veterinary Medical Association and with approval from the Animal Care Committee of the University of Wisconsin–Madison. Humane euthanasia was performed with 390 mg of pentobarbitone injected into the peritoneal cavity at the end of the experiment.

### Experimental design

To determine whether skeletal adaptation to loading of the right ulna, in the form of a modeling response, affected distant long bones through a neuronally regulated pathway, 24 rats were treated with a short period of nonfatiguing cyclic loading at an initial peak strain of −3,750 µɛ. In the block + load group, 8 of these rats received perineural anesthesia of the right brachial plexus before loading (see below), whereas the remaining 16 rats were assigned to the load group. Similarly, to determine systemic skeletal adaptive responses to a more pronounced biophysical stimulus, the right ulna of an additional 16 rats was loaded until 40% loss of stiffness was attained using an initial peak strain of −3,000 µɛ. In the block + fatigue group, 8 of these rats also received perineural anesthesia of the right brachial plexus before fatigue loading, whereas the remaining rats were assigned to the fatigue group. A sham-loaded group of 12 rats was used to validate the results. These rats received the same experimental conditions as the load and fatigue groups but were not loaded.

All procedures were performed under isoflurane-induced general anesthesia, as described previously.(9) For analgesia, butorphanol (0.5 mg/kg) was given by subcutaneous injection 15 minutes before induction of anesthesia and again immediately after loading. Humane euthanasia was performed at the end of the 10-day experimental period. Blood samples were collected from each rat immediately prior to euthanasia in the fatigue group, the block + fatigue group, and the sham group using a heparinized syringe. To label all new bone formation that occurred during the treatment period, rats received two intraperitoneal injections of the fluorochrome calcein at 7 mg/kg (Sigma, St. Louis, MO, USA), the first immediately after loading and a second 7 days after loading.(9,16)

### Anesthesia of the brachial plexus

Five minutes before loading, rats assigned to the brachial plexus blocking groups underwent perineural anesthesia of the nerves of the right brachial plexus using bupivicaine (Marcaine 0.5%, Hospira, Lake Forest, IL, USA) at a dose of 2 mg/kg. An insulated needle was used to make the injection; correct positioning of the needle was confirmed using a nerve stimulator (Micro Stim, Neuro Technology, Houston, TX, USA).(9) Perineural positioning of the needle induced an observable limb movement after activation of the nerve stimulator. Functional blocking was confirmed after recovery from general anesthesia by the presence of right thoracic limb paralysis, which resolved within 2 hours of loading.

### In vivo ulna loading

**Fig. 1:**
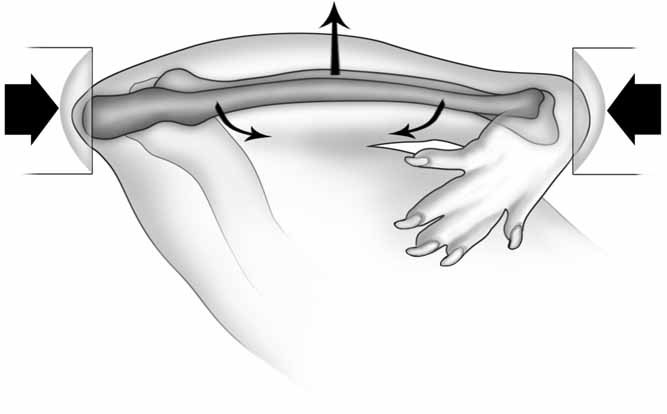
Schematic diagram of the rat ulna loading model. The antebrachium was placed horizontally in loading cups attached to a materials testing machine. The mediolateral diaphyseal curvature of the rat ulna is accentuated through axial compression, most of which is translated into a bending moment, which is greatest at approximately 60% of the total bone length measured from the proximal end of the ulna.(18) Ulna end loading can be used to induce either a modeling response through loading without inducing bone fatigue or a mixed modeling/remodeling response by loading the bone until fatigue occurs; bone fatigue is determined to have occurred when an increase in displacement amplitude occurs during cyclic loading and by histologic evidence of bone microdamage.

All in vivo loading of the right ulna was performed under isoflurane-induced general anesthesia. The right antebrachium of each rat was placed horizontally between two loading cups that are fixed to the loading platen and actuator of a materials testing machine (Model 8800 DynaMight, Instron, Canton, MA, USA) with a 250-N load cell (Honeywell Sensotec, Canton, MA, USA). The right ulna then underwent loading through axial compression, which accentuates the preexisting mediolateral curvature of the diaphysis of the ulna, translating most of the axial force into a bending moment (Fig. 1). The relationship between peak load entered into the materials testing machine control module and the actual force and peak strain applied to the ulna was determined previously.(9) In the load and block + load groups, loading was performed for 1500 cycles at 4 Hz, with an initial peak strain of −3,750 µɛ (−18 N entered into materials testing machine, −16.8 N applied to ulna). In the fatigue and block + fatigue groups, cyclic loading was performed at 4 Hz.(17) Loading was initiated at −16 N, and the load applied to the ulna was increased incrementally until fatigue was initiated. Loading then was terminated when 40% loss of stiffness was attained.

### Bone histomorphometry

The right and left ulnas, humeri, tibias, and femurs were dissected along with surrounding tissue. Bones were wrapped in saline-saturated gauze and stored at −20°C. Before sectioning, bones were dehydrated in a graded series of ethanol (70%, 100%) and embedded in methyl methacrylate. Transverse calcified sections 120 µm thick were made and mounted on standard microscope slides. Ulnas were sectioned at 60% total bone length, measured from the proximal end, where it has been shown that maximal adaptation takes place in this model.(18) All other long bones were sectioned at the mid-diaphysis. Sections were examined using bright-field and confocal microscopy (Bio-Rad MRC-1024 Laser Scanning Confocal Microscope, Bio-Rad, Hercules, CA, USA). Periosteal, endosteal, and total labeled bone areas (Ps.L.B.Ar, Es.L.B.Ar, and Tt.L.B.Ar, %) were determined using standard methods.(9,10,19)

Preliminary work (data not shown) suggested that in young, rapidly growing male rats with a skeleton that is actively modeling (MS/BS and MAR of 73.8 ± 17.9% and 5.4 ± 3.4 µm, respectively),(9) direct quantification of labeled bone provides a more sensitive measurement method for assessing load-induced bone formation when a single colored fluorochrome label is used compared with classic morphometry methods.(9) All measurements were made by a single observer (SS). Data were normalized to the original cortical area to account for minor variations in rat size. In the cyclic fatigue experiment, crack surface density (Cr.S.Dn, µm/mm^2^) was quantified in right and left ulnas as an indicator of fatigue microdamage. Additionally, ulna resorption space number density and resorption space area density were quantified (Rs.N/T.Ar, *n*/mm^2^, Rs.Ar/T.Ar, mm^2^/mm^2^) as indicators of remodeling. Data were normalized to the original cortical area to account for minor variations in rat size.

### Plasma markers of bone turnover

Plasma was isolated from each blood sample after centrifugation and stored at −80°C. Plasma then was analyzed for tumor necrosis factor α (TNF-α), osteoprotegerin (OPG), receptor activator for nuclear factor κβ ligand (RANKL), and tartrate-resistant acid phosphatase 5b (TRAP5b) concentrations using ELISA kits validated for the rat (TNF-α, OPG, RANKL, R&D Systems Minneapolis, MN, USA; TRAP5b, Immunodiagnostic Systems, Ltd., Fountain Hills, AZ, USA).

### Statistical analysis

The Kolmogorov-Smirnov test was used to confirm that data were normally distributed. For analysis of labeled new bone formation, a one-way ANOVA with a Dunnett's post hoc test was used to determine differences from sham control. Repeated-measures ANOVA with a planned comparison post hoc test was used to compare load and block + load groups for both loading regimens. The Kruskal Wallis ANOVA test and the Mann-Whitney *U* test were used to compare Rs.N/T.Ar, Rs.Ar/T.Ar, and Cr.S.Dn with sham control. The Kruskal Wallis ANOVA test and the Mann-Whitney *U* test also were used to compare plasma concentrations of TNF-α, OPG, RANKL, and TRAP5b between groups. Results were considered significant at *p* < .05. Data are reported as mean ± SD or median and range for nonparametric data.

## Results

### Mechanical loading of the right ulna induced modeling responses in multiple long bones

**Fig. 2:**
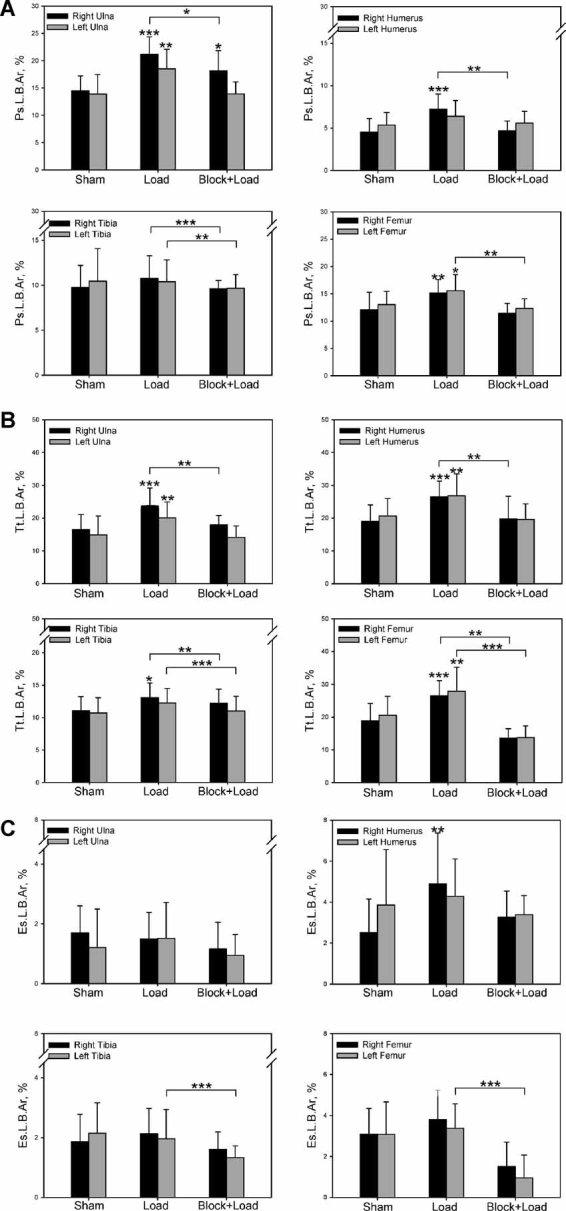
Load-induced bone formation in thoracic and pelvic limb long bones of male Sprague-Dawley rats was influenced by neuronal signaling during bone modeling in response to unilateral cyclic loading of the right ulna. The majority of adaptive bone formation in distant bones and a large proportion of bone formation in the loaded (right) ulna was neuronally regulated. (*A*) Periosteal labeled bone area (Ps.L.B.Ar) in response to mechanical loading was seen in the loaded (right) ulna and numerous long bones that were not loaded. Formation of labeled periosteal new bone was decreased when loading was performed during temporary blocking of neuronal signaling between the right thoracic limb and the spinal cord by anesthesia of the loaded limb's brachial plexus. (*B*) Total labeled bone area (Tt.L.B.Ar) in response to mechanical loading was more evident in pelvic limb bones than in the humeri. (*C*) Endosteal labeled bone area (Es.L.B.Ar) was increased only in the contralateral humerus after right ulnar loading. *p < .05; **p < .01; *** *p* <0.001 versus the relevant sham control. Differences between the load group and the block + load group are also indicated. Error bars represent SD. Sham group *n* = 12; load group *n* = 16; block + load group *n* = 8.

When the load group was compared with the sham group, increased bone formation was found in the loaded right ulna, as expected (*p* < .001, Fig. 2*A*), principally on the periosteal surface and within the cortex of the ulna. In the remaining long bones, Ps.L.B.Ar was increased in 4 of 7 bones (left ulna, right humerus, and both femurs; *p* < .05). Similarly, Tt.L.B.Ar was increased in the loaded right ulna (*p* < .001) and in 6 of the remaining 7 long bones (left ulna, both humeri, right tibia, and both femurs; *p* < .05; Fig. 2*B*). In contrast, Es.L.B.Ar in the right ulna in the loaded and sham groups was not significantly different (Fig. 2*C*) and was increased only in the right humerus (*p* = .003). Although not dramatic, increased bone formation in distant bones was overall more evident in ipsilateral long bones than in contralateral long bones.

**Fig. 3:**
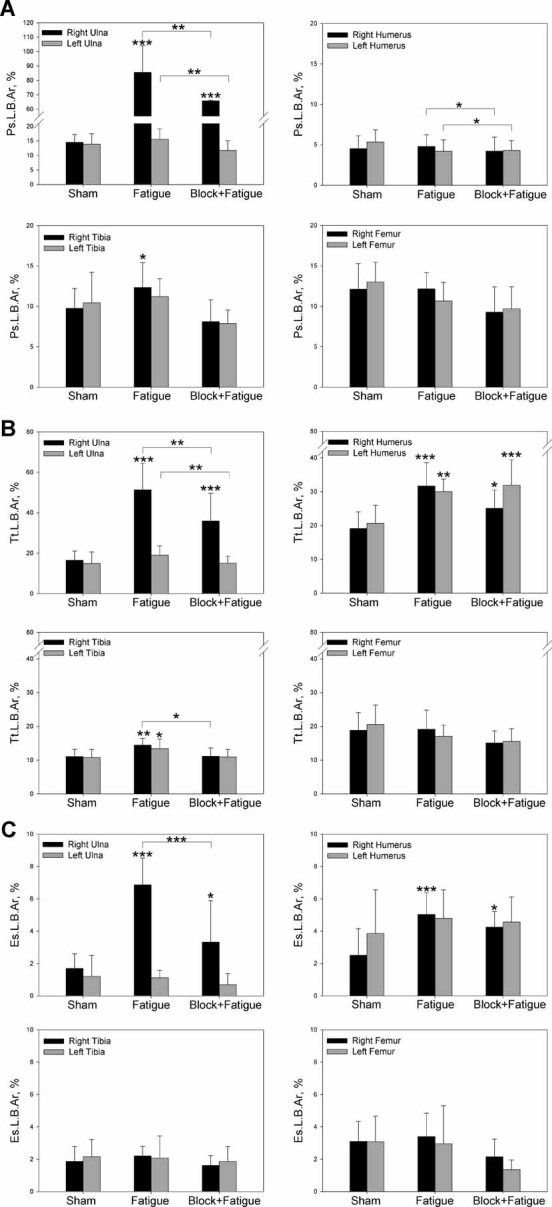
Fatigue loading of the right ulna induced neuronally regulated new bone formation in both thoracic and pelvic limb long bones. (*A*) Periosteal labeled bone area (Ps.L.B.Ar) was decreased significantly in the loaded ulna, the contralateral ulna, and both humeri when loading occurred during brachial plexus anesthesia, which temporarily eliminated neuronal signaling between the right thoracic limb and spinal cord. (*B*) When compared with the sham group, the fatigue group had significantly increased total labeled bone area (Tt.L.B.Ar) in multiple long bones. Brachial plexus anesthesia significantly decreased Tt.L.B.Ar in both ulnas and the contralateral tibia. (*C*) Fatigue loading of the right ulna affected only endosteal new bone area (Es.L.B.Ar) in the right ulna and humerus, with a significant blocking effect occurring in the loaded (right) ulna. *p < .05; **p < .01; ***p < .001 versus the relevant sham control. Differences between the fatigue group and the block + fatigue group are also indicated. Error bars represent SD. Sham group *n* = 12; fatigue group *n* = 8; block + fatigue group *n* = 8.

After cyclic fatigue loading and induction of ulna microdamage, a marked increase in bone formation was found in the loaded right ulna, as expected (*p* < .001; Fig. 3*A*), principally on the periosteal surface. In the remaining long bones, Ps.L.B.Ar was increased in 1 of 7 bones (right tibia). Similarly, Tt.L.B.Ar was increased in the loaded right ulna (*p* < .001) and in 4 of the remaining 7 long bones (both humeri and both tibias; *p* < .05; Fig. 3*B*). Es.L.B.Ar also was markedly increased in the right ulna (*p* < .001; Fig. 3*C*) and in 1 of the remaining 7 long bones (right humerus; *p* < .001). Again, increased bone formation in distant bones was more evident in ipsilateral long bones than in contralateral long bones.

### Brachial plexus anesthesia during cyclic bone loading blocks adaptive modeling responses

**Fig. 4:**
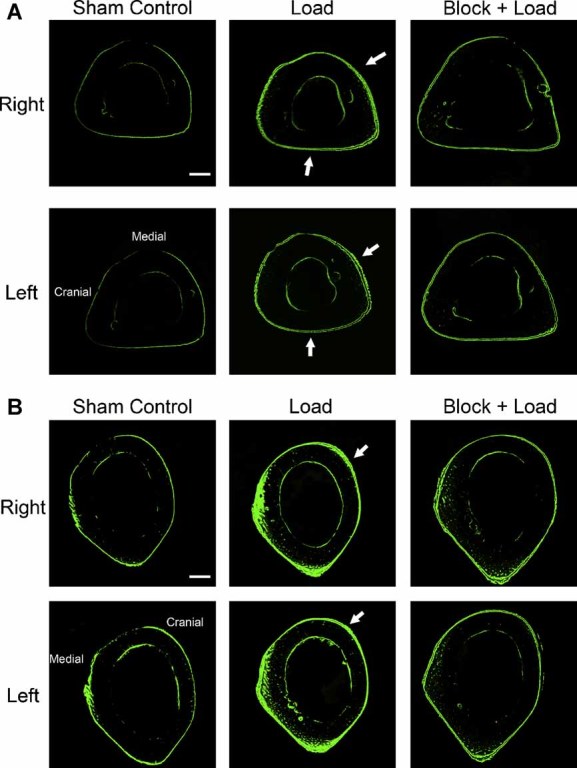
Bone formation in the pelvic limb bones of male Sprague-Dawley rats was increased significantly after cyclic loading of the right ulna. (*A*) Brachial plexus anesthesia of the loaded limb at the time of ulnar loading resulted in decreased new bone formation in both the right (ipsilateral) and left (contralateral) tibias. (*B*) Loading the right ulna resulted in increased bone formation in both the right (ipsilateral) and left (contralateral) femurs that were not loaded. As was seen in the tibias, brachial plexus anesthesia at the time of right ulnar loading eliminated these effects (arrows). Confocal photomicrographs of calcified transverse sections of tibias and femurs at 50% of bone length after 10 days of adaptation to a short period of cyclic loading that was applied to the right ulna (1500 cycles at 4 Hz and an initial peak strain of −3750 µɛ). Load-induced formation was double labeled with calcein. Bars = 500 µm for *A* and 500 µm for *B*. Sham group *n* = 12; load group *n* = 16; block + load group *n* = 8.

Temporary blockade of neuronal signaling between the spinal cord and the loaded limb during mechanical loading of the right ulna ameliorated labeled bone formation in both the loaded bone and other long bones within the skeleton (Fig. 2, Tables 1 and 2). In the block + load group, Ps.L.B.Ar was decreased in the right ulna compared with the load group (*p* < .05; Fig. 2*A*). However, right ulnar Ps.L.B.Ar in the block + load group remained increased compared with the sham group (*p* < .05). In all the remaining long bones, Ps.L.B.Ar in the block + load group and the sham group was not significantly different; Ps.L.B.Ar in the right humerus, both tibias, and the left femur was significantly blocked by brachial plexus anesthesia (*p* < .01; Fig. 4). Similarly, in the block + load group, Tt.L.B.Ar was decreased in the right ulna compared with the load group (*p* < .01; Fig. 2*B*) to the level of the sham group. In 5 of the 6 remaining long bones in which Tt.L.B.Ar was increased in the load group, a significant blocking effect was identified in the block + load group (*p* < .01; Fig. 2, Table 1). For example, in the left ulna of the load group, Tt.L.B.Ar was increased compared with the sham group, whereas in the block + load group, Tt.L.B.Ar was not significantly different from the sham group. Es.L.B.Ar in the left tibia and left femur was decreased in the block + load group compared with the load group (*p* < .001; Fig. 2*C*). In the right humerus, Es.L.B.Ar was increased in the load group compared with sham group, whereas Es.L.B.Ar in the block + load group and the sham group was not significantly different.

**Table 1:** Proportion of Load-Induced Bone Formation Blocked by Brachial Plexus Anesthesia in Rat Long Bones After Cyclic Loading of the Right Ulna

Bone	Sham control (*n* = 12)	Load (−3750 µɛ) (*n* = 16)	Brachial plexus block + load (−3750 µɛ) (*n* = 8)	Blocking proportion (%) above sham control
Ps.L.B.Ar, %				
Right (loaded) ulna	14.50 ± 2.71	21.20 ± 3.17*	18.18 ± 3.67†	45%
Left ulna	13.93 ± 3.63	18.54 ± 3.59‡	13.94 ± 2.16	99%
Right humerus	4.53 ± 1.59	7.25 ± 1.78*	4.69 ± 1.14	94%
Left humerus	5.35 ± 1.50	6.39 ± 1.87	5.58 ± 1.38	78%
Right tibia	9.76 ± 2.45	10.77 ± 2.51	9.61 ± 0.92	>100%
Left tibia	10.45 ± 3.77	10.39 ± 2.43	9.67 ± 1.52	n/a
Right femur	12.10 ± 3.18	15.12 ± 2.47‡	11.48 ± 1.74	>100%
Left femur	13.01 ± 2.45	15.56 ± 2.95†	12.31 ± 1.77	>100%
Tt.L.B.Ar, %				
Right (loaded) ulna	16.54 ± 4.64	23.83 ± 5.38*	17.92 ± 2.93	81%
Left ulna	14.91 ± 5.69	20.08 ± 4.84‡	14.11 ± 3.53	>100%
Right humerus	19.11 ± 4.93	26.53 ± 4.76*	19.79 ± 6.84	91%
Left humerus	20.63 ± 5.38	26.81 ± 6.67‡	19.60 ± 4.73	>100%
Right tibia	11.06 ± 2.17	13.07 ± 2.28†	12.20 ± 2.19	43%
Left tibia	10.71 ± 2.48	12.25 ± 2.25	11.02 ± 2.28	80%
Right femur	18.86 ± 5.26	26.50 ± 4.63*	13.57 ± 2.88	>100%
Left femur	20.56 ± 5.78	27.88 ± 7.38‡	13.79 ± 3.55	>100%
Es.L.B.Ar, %				
Right (loaded) ulna	1.71 ± 0.95	1.50 ± 0.89	1.46 ± 0.86	n/a
Left ulna	1.21 ± 1.29	1.52 ± 1.20	0.95 ± 0.68	>100%
Right humerus	2.50 ± 1.65	4.90 ± 2.47‡	3.25 ± 1.29	69%
Left humerus	3.86 ± 2.70	4.28 ± 1.83	3.40 ± 0.93	>100%
Right tibia	1.87 ± 0.91	2.13 ± 0.84	1.61 ± 0.58	>100%
Left tibia	2.15 ± 1.07	1.96 ± 0.98	1.34 ± 0.40	n/a
Right femur	3.10 ± 1.25	3.80 ± 1.44	1.51 ± 1.19	>100%
Left femur	3.08 ± 1.58	3.38 ± 1.20	0.96 ± 1.11	>100%

Data represent mean ± SD. >100% indicates brachial plexus blocking suppressed bone formation below sham control.

Blocking proportion (%) = [1 – (block + load group – sham control group)/(load group – sham control group)] × 100%.

† *p* < .05

‡ *p* < .01

* *p* < .001 versus the sham control.

Ps.L.B.Ar = periosteal labeled bone area; Tt.L.B.Ar = total labeled bone area; Es.L.B.Ar = endosteal labeled bone area; n/a = not applicable because loaded mean was below sham control.

**Table 2:** Proportion of Load-Induced Bone Formation Blocked by Brachial Plexus Anesthesia in Rat Long Bones After Cyclic Fatigue Loading of the Right Ulna

Bone	Sham control (*n* = 12)	Fatigue (*n* = 8)	Brachial plexus block + fatigue (*n* = 8)	Blocking proportion (%) above sham control
Ps.L.B.Ar, %				
Right (loaded) ulna	14.50 ± 2.71	85.47 ± 18.49*	65.66 ± 14.12*	28%
Left ulna	13.93 ± 3.63	15.57 ± 3.59	11.70 ± 3.33	>100%
Right humerus	4.53 ± 1.59	4.80 ± 1.44	4.22 ± 1.74	>100%
Left humerus	5.35 ± 1.50	4.19 ± 1.43	4.29 ± 1.22	9%
Right tibia	9.76 ± 2.45	12.34 ± 3.06^†^	8.10 ± 2.71	>100%
Left tibia	10.45 ± 3.77	11.19 ± 2.20	7.87 ± 1.68	>100%
Right femur	12.10 ± 3.18	12.17 ± 1.99	9.29 ± 3.12	>100%
Left femur	13.01 ± 2.45	10.65 ± 2.30	9.70 ± 2.72	n/a
Tt.L.B.Ar, %				
Right (loaded) ulna	16.54 ± 4.64	51.38 ± 13.04*	35.94 ± 13.76*	44%
Left ulna	14.91 ± 5.69	19.40 ± 4.54	15.10 ± 3.41	96%
Right humerus	19.11 ± 4.93	31.69 ± 6.88*	25.09 ± 5.37†	52%
Left humerus	20.63 ± 5.38	29.99 ± 3.74‡	31.96 ± 7.47*	n/a
Right tibia	11.06 ± 2.17	14.41 ± 2.04‡	11.12 ± 2.52	98%
Left tibia	10.71 ± 2.48	13.40 ± 2.87†	10.97 ± 2.17	90%
Right femur	18.86 ± 5.26	19.12 ± 5.70	15.12 ± 3.54	>100%
Left femur	20.56 ± 5.78	17.07 ± 3.33	15.52 ± 3.84	n/a
Es.L.B.Ar, %				
Right (loaded) ulna	1.71 ± 0.95	6.86 ± 1.66*	3.33 ± 2.56†	68%
Left ulna	1.21 ± 1.29	1.13 ± 0.46	0.69 ± 0.69	>100%
Right humerus	2.50 ± 1.65	5.03 ± 1.35*	4.25 ± 0.97†	31%
Left humerus	3.86 ± 2.70	4.79 ± 1.76	4.57 ± 1.54	24%
Right tibia	1.87 ± 0.91	2.21 ± 0.58	1.62 ± 0.61	>100%
Left tibia	2.15 ± 1.07	2.08 ± 1.36	1.87 ± 0.91	n/a
Right femur	3.10 ± 1.25	3.40 ± 1.45	2.15 ± 1.09	>100%
Left femur	3.08 ± 1.58	2.96 ± 2.35	1.36 ± 0.59	n/a

Data represent mean ± SD. >100% indicates that brachial plexus blocking suppressed bone formation below sham control.

Blocking proportion (%) = [1 – (block + fatigue group – sham control group)/(fatigue group – sham control group)] × 100%.

† *p* < .05

‡ *p* < .01

* *p* < .001 versus the sham control.

Ps. L.B.Ar = periosteal labeled bone area; Tt.L.B.Ar = total labeled bone area; Es.L.B.Ar = endosteal labeled bone area; n/a = not applicable because loaded mean was below sham control.

**Fig. 5:**
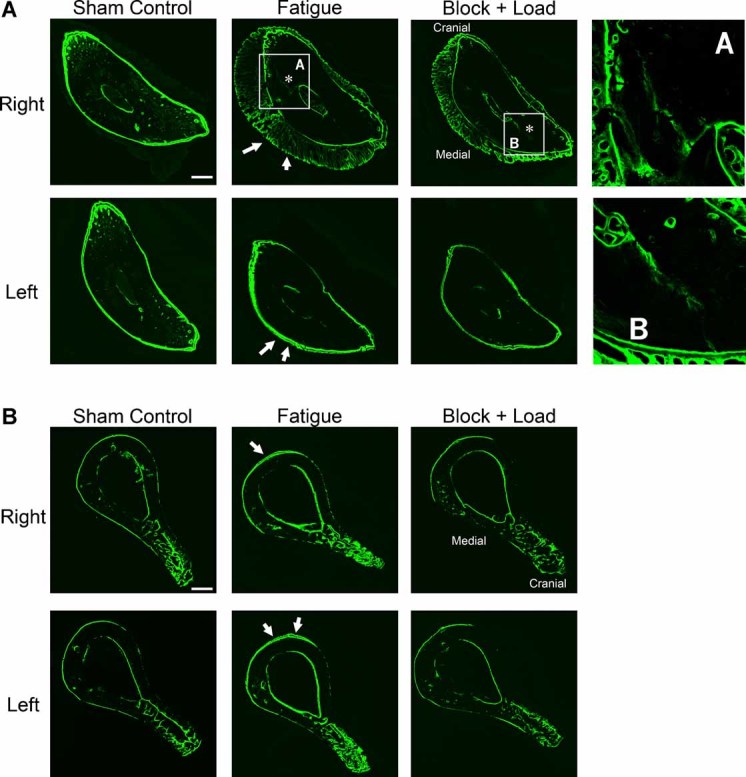
Cyclic fatigue loading of the right ulna induces adaptive bone formation that is neuronally regulated in multiple thoracic limb bones in male Sprague-Dawley rats. (*A*) Labeled bone formation was decreased in both the right (loaded) and left (contralateral) ulnas when loading was performed during temporary blocking of neuronal signaling between the right thoracic limb and the spinal cord by anesthesia of the loaded limb's brachial plexus, although bone formation in the loaded ulna was not completely blocked. Microcracking of the caudomedial region of the right (loaded) ulna can be appreciated (*). (*B*) Fatigue loading of the right ulna also resulted in increased labeled bone formation in the right (ipsilateral) and left (contralateral) humeri that were not directly loaded. Periosteal labeled bone formation was significantly attenuated when the brachial plexus was anesthetized during right ulnar loading (*arrows*). Confocal photomicrographs of calcified transverse sections of ulna at 60% of bone length, from proximal to distal, and humeri at 50% of bone length 10 days after the right ulna was fatigue loaded to 40% loss of stiffness. Load-induced bone formation was double labeled with calcein. Bars = 250 µm for *A* and 500 µm for *B*. Sham control group *n* = 12; fatigue group *n* = 8; block + fatigue group *n* = 8.

In the block + fatigue group, Ps.L.B.Ar, Tt.L.B.Ar, and Es.L.B.Ar also were decreased in the fatigue-loaded right ulna compared with the fatigue group (*p* < .006; Fig. 3) but not to the level of the sham group (Table 2, Figs. 3*A* and 5*A*). Similarly, the blocking proportion in the right humerus also was lower than in block + fatigue group (Table 2). Decreased Ps.L.B.Ar was observed in 3 of the remaining 7 long bones (*p* < .05; Fig. 3*A*) in the block + fatigue group compared with the fatigue group (left ulna and both humeri; Figs. 3*B* and 5*B*). Decreased Tt.L.B.Ar was observed in 2 of the remaining 7 long bones in the block + fatigue group compared with the fatigue group (left ulna and right tibia; *p* < .05; Fig. 3*B*). Tt.L.B.Ar was increased in both humeri of the block + fatigue group compared with the sham group (*p* < .05). Brachial plexus anesthesia did not have a significant blocking effect on Es.L.B.Ar in bones other than the fatigue-loaded right ulna (Fig. 3*C*).

### Brachial plexus anesthesia during cyclic bone fatigue did not block adaptive remodeling

**Fig. 6:**
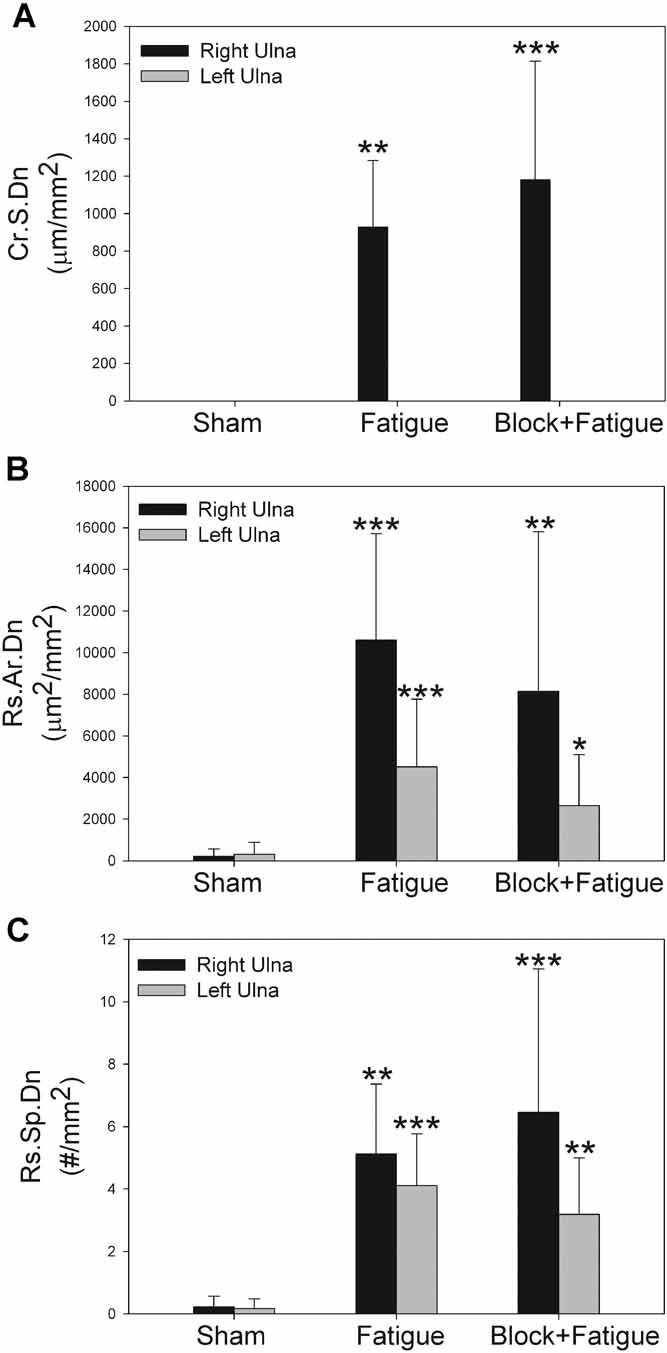
Cyclic fatigue loading of the right ulna resulted in microcrack formation in the right ulna and increased resorption space area density (Rs.Ar.Dn) and resorption space number density (Rs.Sp.Dn) in both the right and left ulnas compared with the sham control. Brachial plexus anesthesia did not affect any of these parameters in either the right (fatigue-loaded) ulna or the left (contralateral) ulna, which was not loaded. *p < .05; **p < .01; ***p < .001 versus the relevant sham control. Error bars represent SD. Sham group *n* = 12; fatigue group *n* = 8; block + fatigue group *n* = 8.

Cr.S.Dn was increased in the right ulnas of both the fatigue and the block + fatigue groups compared with the sham group (*p* < .002; Fig. 6*A*). No microcracks were found in the left ulnae. Rs.Sp.Dn and Rs.Ar.Dn were increased in loaded and contralateral ulnas of both the fatigue and block + fatigue groups compared with the sham group (*p* < .001 and *p* < .002 respectively; Fig. 6*B, C*). Significant blocking effects from brachial plexus anesthesia on Rs.Sp.Dn and Rs.Ar.Dn were not found.

### Effect of cyclic bone fatigue and brachial plexus anesthesia on plasma markers of bone remodeling

TNF-α and RANKL were not detectable in the plasma in the sham, fatigue, and block + fatigue groups at 10 days. OPG was detectable in some rats, but differences were not significant between groups (*p* > .5). TRAP5b was consistently detected in the fatigue group and was significantly decreased in the block + fatigue group to the level of the sham control (*p* = .01; Table 3).

**Table 3:** Effect of Brachial Plexus Anesthesia on Serum Markers of Bone Remodeling After Cyclic Fatigue Loading of the Right Ulna in Male Rats

Group	TNF-α (pg/mL)	RANKL (pg/mL)	OPG (pg/mL)	TRAP5b (U/L)
Sham (*n* = 6)	0 (0, 0)	0 (0, 0)	0 (0, 14.5)	0 (0, 0)
Fatigue (*n* = 7)	0 (0, 0)	0 (0, 0)	0 (0, 18.5)	2.2 (1.4, 3.4)*
Block + fatigue (*n* = 7)	0 (0, 0)	0 (0, 0)	0 (0, 0)	0 (0, 0)

Data represent median (range). TNF-α = tumor necrosis factor; RANKL = receptor activator of Nf-κB ligand; OPG = osteoprotegerin; TRAP5b = tartrate-resistant acid phophatase 5b.

* *p =* .01 versus other two groups.

## Discussion

Functional adaptation of the skeleton to mechanical loading for many years has been considered primarily a local phenomenon. In this study we used the rat ulna end-loading model to induce either a modeling response without fatigue damage or a mixed modeling/remodeling response associated with induction of bone fatigue by cyclic loading. We showed that a single period of loading applied to a single long bone (the right ulna) results in increased bone formation in long bones of both the thoracic and pelvic limbs that were not loaded. This new bone formation appears to be neuronally regulated through a crosstalk mechanism via the central nervous system because brachial plexus anesthesia had significant blocking effects on load-induced bone formation in multiple bones, and these changes generally were not associated with detectable changes in plasma markers of bone metabolism.

It has been recognized that the acquisition of relatively small amounts of cortical bone dramatically increases skeletal fatigue resistance. Therefore, although the increases in bone formation at distant skeletal sites are relatively small compared with the formation seen in the loaded bone, they may contribute substantially to improved skeletal strength and fatigue resistance.(20) Although not addressed in this study, it would be relevant in future studies to determine whether these distant long bones have altered biomechanical properties.

Brachial plexus anesthesia with bupivicaine was used in this study to induce temporary blockade of neuronal signaling to and from the loaded limb during bone loading.(9) Brachial plexus anesthesia with bupivicaine will block signaling in both sensory and motor innervation between the spinal cord and the limb and thus anesthetize the limb distal to the elbow and block neuronal signaling between the loaded ulna and the spinal cord.(21) Brachial plexus anesthesia may not completely block innervation of the upper limb and the humerus. Our results suggest that the increased bone formation seen in distant long bones that were not loaded was mainly influenced by neuronal signaling events that occur at the time of mechanical loading. Interestingly, the loading regimen that did not induce fatigue of the loaded bone resulted in more obvious modeling effects in distant long bones than did the fatigue-loading regimen. A large proportion of adaptive new bone formation in the distant long bones was inhibited by brachial plexus anesthesia of the loading limb at the time of loading. In contrast, a smaller proportion of bone formation in the loaded limb was inhibited by brachial plexus anesthesia. This proportion was lower when a more intense biophysical stimulus was provided through use of cyclic fatigue loading, indicating that adaptive responses to mechanically induced signaling events in loaded bones are controlled by both neuronal and nonneuronal pathways. Collectively, these observations suggest that when a modeling response is induced locally, the entire skeleton undergoes an adaptive response, presumably to optimize skeletal mass and architecture in response to changes in the skeletal loading environment.

OPG, RANKL, and TRAP5b are all markers of bone metabolism and remodeling.(22,23) The lack of a difference in OPG and RANKL plasma concentrations between the fatigue group, the block + fatigue group, and the sham group plasma 10 days after fatigue loading may be due to the length of time that had lapsed since the loading episode because formation of resorption spaces may occur as early as a few days after bone loading in this model. TRAP5b plasma concentrations, however, were more interesting; an increase in plasma TRAP5b was seen in the fatigue group, whereas plasma concentrations in the block + fatigue group were the same as in the sham group. TRAP5b is expressed on both immature and mature osteoclasts; plasma TRAP5b concentrations are proportional to osteoclast number.(24) Therefore, this observation suggests that neuronal signaling, in addition to signaling via osteocyte apoptosis,(25,26) may be important for recruitment and activation of osteoclasts for targeted remodeling of bone microdamage. It is interesting also to note that formation of resorption spaces in the contralateral ulna also was significantly increased relative to the sham group, although there was not a significant blocking effect on Rs.Ar.Dn and Rs.Sp.Dn. The role of neuronal signaling in the recruitment of osteoclasts for targeted remodeling requires further investigation. Our data suggest that targeted remodeling may involve a synergy between neuronal and nonneuronal signaling. In future work, analysis of bone marrow cultures from loaded and nonloaded bones for expression of TRAP5b and other markers of osteoclastogenesis may be informative.

TNF-α is thought of primarily as an inflammatory mediator, although recent studies have indicated that it is a part of the pathophysiology of a number of disease conditions, including osteoporosis.(27) However, in this study TNF-α was not detectable in plasma in any group, suggesting that it is unlikely that TNF-α signaling is contributing to skeletal metabolism in this model.

Osteocytes have long been considered the regulators of mechanically induced signaling events in bone,(28) and a widely held view currently is that local strain-related effects are the basis for functionally adaptive modeling responses to skeletal loading events.(5,10,29) The concept that skeletal responses to bone loading may involve physiologic responses in regions of the skeleton that were not loaded or may be neuronally regulated is controversial. Historically, many functional adaptation studies have been conducted using the contralateral limb as a control, and thus distant effects of loading may not have been identified. In recent work using tibial loading using C57/Bl6 female mice, adaptive responses to loading were found to be limited only to the loaded bone.(10) There are several potential explanations for this apparent paradox. Mechanosensitivity is known to be influenced by genetic background.(30) Skeletal adaptation is also highly dependent on the nature of the mechanical stimulus. Variations in strain rate, peak strain, loading periodicity, and whether tissue injury is induced during loading all potentially may influence the physiologic response to bone loading.(10) The gender, species, and age of the animals used for experimental studies also may influence skeletal response to bone loading. Young, rapidly growing male rats were used in this study, and modeling effects in distant skeletal sites in response to bone loading may be more evident in young males versus older females. Collectively, recent observations in this field suggest that multiple mechanisms may regulate skeletal responses to mechanical loading. Physiologic signaling pathways that control strain-related functional adaptation, which leads to local adaptive changes in bone architecture, may be distinct from the mechanisms that regulate wider responses in the skeleton to mechanical stimuli.(10) Fundamental understanding of these events likely will hinge on improved knowledge regarding the mechanically induced cell signaling events that regulate skeletal responses to mechanical stimuli and the key factors that lead to activation of wider skeletal responses to bone loading. Functional neuronal connections between different regions of the skeleton do exist.(15) However, the relevance of these connections to skeletal physiology remains to be determined. Such knowledge likely will further understanding of the pathogenesis of skeletal disease.

The data from this study challenge this long-held view and suggest that the regulation of load-induced adaptation is regulated, in part, neuronally and involves changes throughout the appendicular skeleton, including bones that were not loaded. It is now well established that the nervous system has important regulatory influences on skeletal metabolism.(31–33) Additionally, a number of recent studies support the hypothesis that the nervous system may be involved in the regulation of functional adaptation. For example, pharmacologic sympathectomy hinders bone mass acquisition in young, growing rats,(34) and the nervous system has been shown to respond to skeletal loading events and regulate load-induced bone formation.(9,15) This study is an expansion of the latter finding.

Given what is known about the neuroanatomy of bone, the concept that the central nervous system is involved in skeletal adaptation should not be surprising. It is well established that the periosteal envelope of bone is densely innervated with sensory peptidergic fibers and sympathetic fibers(13) and that periosteal nerve fibers are arranged in a meshlike structure that is optimized for the detection of mechanical distortion.(12) Bone cells themselves have been shown to have direct connections with the nervous system through unmyelinated sensory neurons.(14) Through the use of transynaptic viral tracing with attenuated Bartha pseudorabies virus (PRV), anatomic connections have been shown to exist between the distal appendicular skeleton and the brain.(35) A recent study from our laboratory using PRV tracing also has shown that direct neuroanatomic connections exist between limbs and that circuit remodeling enhances these connections at 10 days after unilateral ulnar loading(15); this study shows that the limbs are directly connected through the spinal cord and that the spinal cord exhibits physiologic responses to skeletal loading events. Interneurons in propriospinal pathways may be principally responsible for this plasticity.(15)

Skeletal innervation also appears to exhibit neuronal plasticity; 10 days after unilateral bone loading, neuropeptide concentrations in both loaded and distant bones are persistently altered.(9) The existence of plasticity in the innervation of the skeleton suggests a potential pathway through which accommodation of the skeleton to habitual loading may occur.(36,37) Thus it would be of interest to determine whether skeletal accommodation to habitual loading could be modified by brachial plexus anesthesia during repeated bouts of bone loading.

There are a number of limitations associated with this study. We chose to perform ulna loading at −18 N and 4 Hz for 1500 cycles. Because of viscoelastic effects from overlying soft tissues, the actual amount of load applied to the rat ulna was approximately −16.8 N rather than the −18 N that was entered into the materials testing machine.(38) It has been shown previously that when using an end-loading in vivo model, increasing the frequency of cycles results in a decreased amount of load being applied to the ulna, again because of viscoelastic effects.(38) Thus the loading protocol used for this study is similar to those used in other studies in this field, for example, use of −17 N and 2 Hz.(39–41) Our strain-gauge measurements, taken at 60% of total ulnar length when measured from the proximal end, indicated that the amount of load entered (−18 N) resulted in approximately −3750 µɛ; although this strain is slightly higher than what has been reported by other laboratories with similar loading protocols, the difference may be due to our placement of the strain gauge at the level of the ulna that experiences the greatest bending moment when loaded as opposed to other laboratories that traditionally have placed the strain gauge at the mid-diaphysis of the ulna.(18,42) Another limitation is that we used only a single fluorochrome color for double labeling. In future work it may be advantageous to use fluorochromes of two different colors to more clearly assess labeled bone formation in the early (0 to 7 days) and late (7 to 10 days) study periods because this should allow for more detailed evaluation of double-labeled bone surfaces that are in close proximity to each other.(10) It also would be interesting to study neuronal regulation of load-induced bone formation with aging and in intact and gonadectimized male and female rats.

We found some variation in the extent of the inhibition of bone formation in various long bones after ulna loading and brachial plexus anesthesia. A blocking effect was least evident in the ipsilateral (right) humerus, particularly after fatigue loading. This likely reflects the fact that brachial plexus anesthesia may not completely anesthetize the upper limb, and thus neuronal signaling to the humerus was not fully blocked. It also should be noted that use of ulna end loading is limited to studies of cortical adaptation of mechanical loading. Other bone-loading models, such as the mouse tibial loading model, could enable study of trabecular adaptation as well to mechanical loading.(10)

The finding that bone loading results in modeling effects in distant skeletal sites is provocative. However, most of the previous studies in this field have used the contralateral limb as a control, and thus modeling effects in the contralateral limb would not necessarily be detected. In this study, we also initiated fluorochrome labeling immediately after bone loading to facilitate detection of early changes in adaptive bone formation after loading.

There is a growing body of evidence that the central nervous system has important regulatory effects on skeletal metabolism.(31,32,43) Data from this study suggest that functional adaptation to a single period of bone loading involves other appendicular long bones in both thoracic and pelvic limbs and that systemic effects on the skeleton from loading are mediated principally via a neuronal crosstalk mechanism between limbs. The sensory innervation of the skeleton exhibits plasticity in response to bone loading. This plasticity most likely occurs via circuit remodeling within the spinal cord.(15) We currently hypothesize that the sensory innervation of bone is capable of acting as both an afferent and an efferent neuronal circuit. Although the specific neurotransmitters involved in this putative pathway are unclear, sex steroids appear to have an important regulatory influence on the sensory innervation to bone(44) and thus may influence functional adaptation of the skeleton through a neuronal pathway.
